# LRP-1 receptor combines EGFR signalling and eHsp90α autocrine to support constitutive breast cancer cell motility in absence of blood supply

**DOI:** 10.1038/s41598-022-16161-y

**Published:** 2022-07-14

**Authors:** Cheng Chang, Xin Tang, Daniel Mosallaei, Mei Chen, David T. Woodley, Axel H. Schönthal, Wei Li

**Affiliations:** 1 Department of Dermatology and the Norris Comprehensive Cancer Centre, University of Southern California Keck Medical Centre, Los Angeles, CA 90033 USA; 2 Department of Molecular Microbiology and Immunology, University of Southern California Keck Medical Centre, Los Angeles, CA 90033 USA

**Keywords:** Cancer, Medical research

## Abstract

Tumor cells face constant stress of ischemic (nutrient paucity and hypoxia) environment when they migrate and invade too fast to outgrow the nearest blood vessels. During the temporary loss of support from circulation, the tumor cells must act self-sufficient to survive and then to migrate to re-connect with the nearest blood supply or die. We have previously reported that ablation of the low-density lipoprotein receptor-related protein 1 (LRP-1) completely nullified the ability of tumour cells to migrate and invade under serum-free conditions in vitro and to form tumours in vivo. The mechanism behind the important function by cell surface LRP-1 was not fully understood. Herein we show that LRP-1 orchestrates two parallel cell surface signalling pathways to support the full constitutive tumour cell migration. First, LRP-1 stabilizes activated epidermal growth factor receptor (EGFR) to contribute half of the pro-motility signalling. Second, LRP-1 mediates secreted Hsp90α autocrine signalling to bring the other half of pro-motility signalling. Only combined inhibitions of the EGFR signalling and the eHsp90α autocrine signalling led to the full blockade of the tumour cell migration as the LRP-1 depletion did. This finding uncovers a novel mechanism by which certain breast cancer cells use LRP-1 to engage parallel signalling pathways to move when they lose contact with blood support.

## Introduction

Cells inside a growing tumour are exposed to an ischemic gradient ranging from efficient nutrients and oxygenation near the stroma to decreasing gradually to near anoxia bordering the necrotic regions of the tumour. When a tumour expands too fast by outgrowing the nearby neovascularization over 150 micron, the tumour cells encounter insufficient O_2_ and nutrient supply. Under these conditions, the tumour cells have two choices. First, switching to a self-sufficient mechanism to survive and to migrate toward the nearest blood vessel. Second, if the cells do not succeed on re-connection with the circulation on time, the ischemic tumour cells will undergo apoptosis and die^1^. Under this life and death circumstance, moving out the stress environment for the tumour cells is the priority. While different tumours may have adapted different self-saving mechanisms under ischemic environment, our current understanding of these mechanisms remains little beyond speculations.

Low density lipoprotein receptor-related protein 1 (LRP-1), also known as alpha-2-macroglobulin receptor (A2MR), apolipoprotein E receptor (APOER) or cluster of differentiation 91 (CD91), is a member of the LDL receptor superfamily found in the plasma membrane of cells that includes at least 11 structurally related members (LDL receptor, LRP1/TβRV, LRP-1b, LRP2/Megalin, VLDL receptor, LRP-3, LRP4/MEGF7, LRP5, LRP6, ApoE receptor 2/LRP8, LRP-9)^2^. Among them, LRP-1 is the only member of the LDL receptor superfamily widely expressed in most cell types studied. Following transcription and translation, the encoded large preproprotein of LRP-1 is proteolytically processed to generate a 515-kDa extracellular and an 85-kDa intracellular subunits that are non-covalently bound to form the mature LRP-1 receptor at cell surface. LRP-1 is functionally diverse as historically a scavenger receptor for endocytosis and as more recently a *bona fide* receptor to bind a wide range of structurally and functionally diverse extracellular ligands for signalling, most noticeably the Akt, NF-κB and Erk1/2 pathways, by which LRP-1 regulates tissue inflammatory reaction, tissue remodelling and cell survival during tissue injury^3^. LRP-1 gene knockout caused embryonic lethality in mice^4^.

LRP-1 plays a critical role in tumour cell migration and invasion in vitro^4–7^ and tumour formation in vivo^8^. Bu’s group showed that miR-205 down-regulates the expression of LRP-1 and causes decreased migration of several tumour cell lines via MMP2 and MMP9^9,10^. Berquand et al. reported that LRP-1 plays a crucial role in MDA-MB-231 cell migration by modulating the membrane extension dynamic^11^. Apert-Collin and colleagues showed that LRP-1 silencing leads to reorganizations of the actin-cytoskeleton by inhibiting FAK activation, activating RhoA activity, MLC-2 phosphorylation and blockade of cell migration using a 3-D configuration assay^12^. Deve’s group reported that LRP-1 regulates cancer-signalling events to support tumour vascular morphology and functionality during angiogenesis^13^. Isaacs’ group showed a novel crosstalk mechanism involving eHsp90-LRP1 dependent regulation of EphA2 function, in which the eHsp90-LRP1 signalling axis regulates AKT signalling and EphA2 activation during glioblastoma cell invasion^14^. We have shown that downregulation of LRP-1 blocks human triple negative breast cancer cell, MDA-MB-231, migration under serum-free conditions in vitro^7^ and tumour formation at mammary fat pad and lung colonization in a mouse xenograft model^8^. However, no study has addressed the important issue of how tumor cells continue their migration and invasion when they lose blood factor support.

In the current study, we have focused our investigation specifically on how cell surface LRP-1 takes over to support tumour cell migration under the loss of environmental support, reminiscent to lack of support from the blood circulation during tumour invasion and metastasis. The tumour cell model for this study is the triple negative breast cancer cells, MDA-MB-231 (ATCC, CRM-HTB-26), one of the highest used tumour cell models in vitro and in vivo (appeared in the titles of over 18,000 publications by the end of 2021). The normal cell control is human (female) breast epithelial cell line, HBL-100 (LINCS, ID:51117). While the mechanism for different tumours with distinct intracellular tumour driver genes likely varies and there will be unlikely one universal mechanism, this study reveals a prototype mechanism for this previously overlooked and important event during tumorigenesis: the LRP-1 receptor coordinates two independent signalling pathways to achieve the single purpose of supporting tumour cell migration under stress.

## Results

### Self-supported breast cancer cell motility versus serum-dependent breast epithelial cell motility

**Figure 1:**
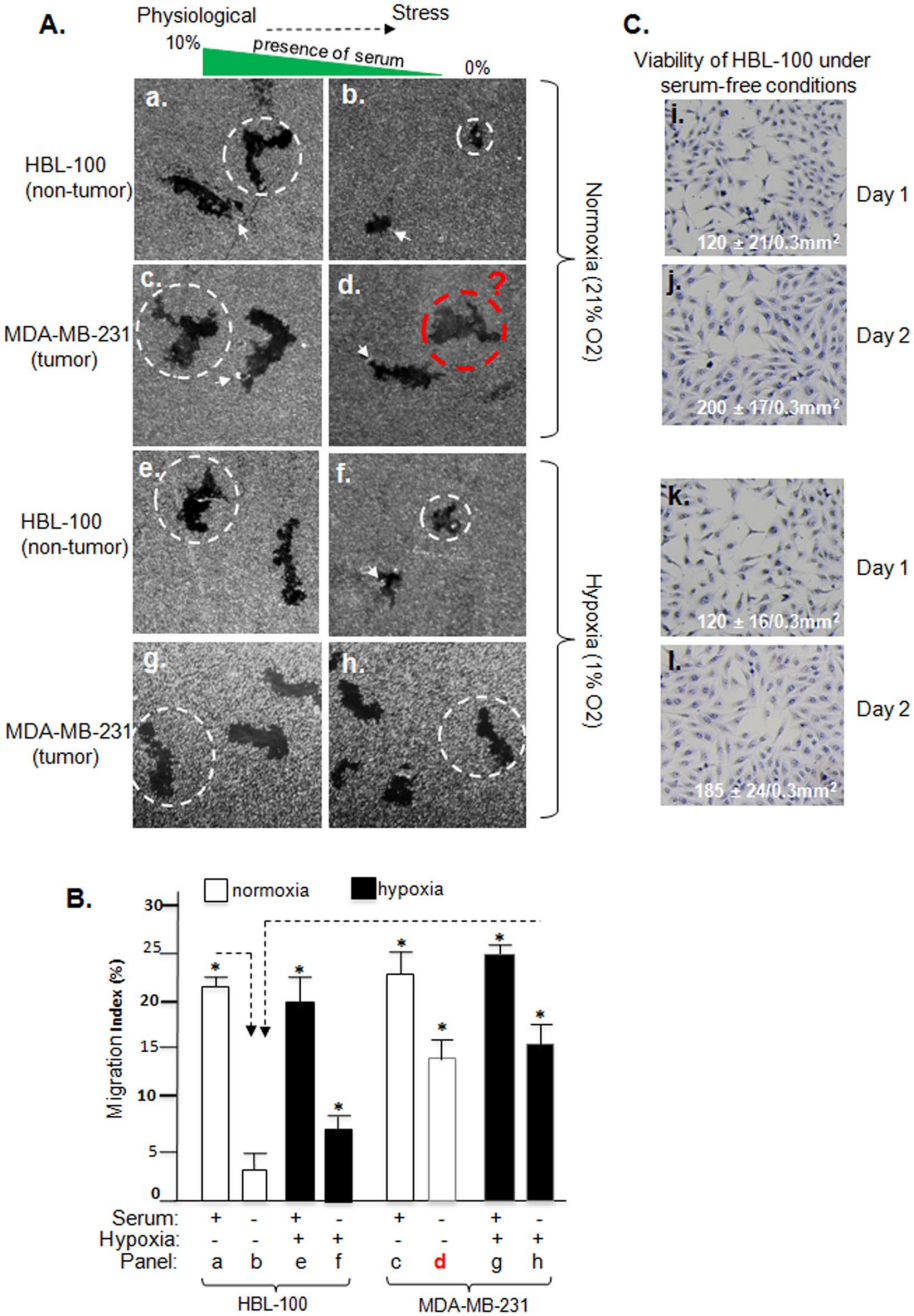
Self-supported constitutive motility of tumour, but not normal, cells. MDA-MB-231 (231) and HBL-100 cells, cultured for 3 days in complete medium with 10% FBS to sub-confluence, were serum starved for 24 h (Day 1). Cells were lifted by trypsin, counted and subjected to colloidal gold migration assay (“Materials and methods”) in medium with or without 10% FBS or hypoxia (1% O2) for additional 16 h at 37 °C (day 2). (**A**) Images of the representative cell migration tracks under indicated conditions (dotted circles indicate average size of the tracks). (**B**) Computer-assisted quantitation of 15 randomly selected microscopic images from each experimental condition as Migration Index (%) (“Materials and methods”). Asterisk indicates significant difference in reference to the control (column b). (**C**) Crystal Violate staining duplicate sets of the same cells under serum-free conditions at the start (day 1) and finish (day 2) of the migration assay under either normoxia (panels i,j) or hypoxia (panels k,l), after washing off dead cells. These results were confirmed by at least three independent experiments with statistical significance.

Constitutive motility even in the absence of blood supply is a critical ability for tumour cells to achieve continued invasion and metastasis^15^. To investigate the mechanism of this intrinsic cancer cell property, we first defined the so-called “self-supported and constitutive motility” of tumour cells by comparing the highly malignant human breast cancer cell line, MDA-MB-231 (https://www.atcc.org/products/htb-26), with the non-tumorigenic human epithelial cell line, HBL-100 (https://lincs.hms.harvard.edu/db/cells/51117/). We utilized the colloidal gold migration assay, arguably the most sensitive and accurate cell migration assay that detects and quantitates single cell motility under any given condition by a darkfield microscope with assistance from a computer software, giving rise to a statistical sum of the so-called “migration Index” (%) of (“Materials and methods”)^16–18^. As shown in Fig. 1A, each migration track (black area) represents the migration trail of a single and serum-starved (16 h) cell under indicated environmental conditions. For example, in the absence of serum factor support, the non-cancer HBL-100 cells were entirely unable to migrate (panel b, indicated by white dotted circle). In contrast, exactly the same and a duplicate set of the cells migrated to create large tracks only when 10% FBS was added to the medium (panel a). Under the full support of serum factors, MDA-MB-231 cells also migrated to create large tracks (panel c), as expected. However, the detected motility from MDA-MB-231 cells was largely self-supported and serum-independent, since it was only slightly reduced under serum-free conditions (emphasized by red dotted circle, panel d). The contribution of hypoxia (1% O_2_) to cell motility was limited to both normal (panels e,f) and tumour (panels g,h) cells, where cell migration remained largely unchanged, in comparison to the effect of serum-free versus serum. Therefore, we mainly focus on serum-free conditions to mimic the lack of blood supply to the tumour cells in vivo for rest of the experiments, unless it becomes necessary controls. Computer-assisted quantitation of randomly selected 15 independent images of the cell migration on collagen-coated colloidal gold surface under each of the indicated conditions is shown in Fig. 1B. The data clearly indicate that MDA-MB-231 cells have acquired a self-supported motility under ischemia-like conditions. The lack of constitutive migration of HBL-100 cells under serum-free conditions was not due to a cell health issue, since crystal violet staining showed not only viability of the cells at the start (day 1, panels i,k) and even an increased cell number at the end of the experiment (day 2, panels j,l) under either normoxia or hypoxia (Fig. 1C). For rest of experiments, we use “231 cells” as the abbreviation of MDA-MB-231 cells.

### Presence of LRP-1 supports the full constitutive tumour cell motility

**Figure 2:**
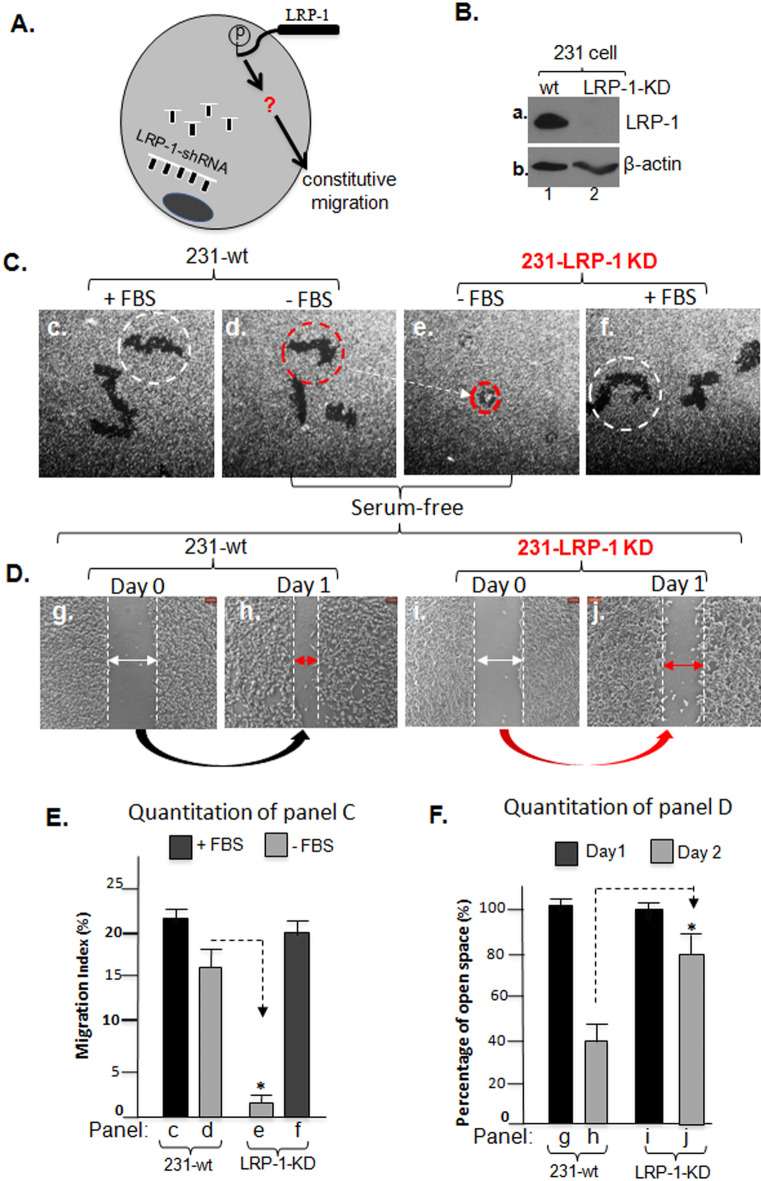
LRP-1 controls the full scale of constitutive motility of MDA-MB-231 tumour cells. (**A**) A schematic illustration of our hypothesis that the LRP-1 receptor controls the constitutive motility of the tumour cells. (**B**) Western blot of total cell lysates shows lentivirus-delivered shRNA down-regulation of LRP-1 in 231 cells. (**C**) Representative images of wild type and LRP-1-downregulated cell migration by colloidal gold migration assay, in which dotter circles indicate average size of migrated tracks under indicated conditions. (**D**) Representative images of wild type and LRP-1-downregulated cell migration by “scratch” assay. Dotted lines indicate the relative open and cell-free space under indicated conditions. (**E**) Computer-assisted quantitation of the cell migration shown in C as Migration Index (%) (“Methods). Asterisk indicates significant reduction in motility versus its corresponding control. (**F**) Quantitation of the average cell-free area (open space) shown in panel (**D**) as the % of the open area on day 0 (100%). Asterisk indicates significant reduction in closing the space versus its corresponding control on day 0. This experiment was repeated multiple times with reproducible results.

The previous findings by many laboratories on LRP-1’s support for tumour cell migration, invasion and tumour formation^7,8,11–14^ led us to investigate the role of LRP-1 in maintaining the constitutive motility and the possible mechanism in 231 cells, as schematically depicted in Fig. 2A. We used lentiviral infection-mediated shRNA delivery to downregulate LRP-1. The efficiency of LRP-1 down-regulation in the cells is shown in Fig. 2B (panel a, lane 2 vs. lane 1) and the specificity or possible off-target concern for the shRNA-LRP-1 is addressed in a later section (see Fig. 5). When the LRP-1-downregulated 231 cells were subjected to colloidal gold migration assay, as shown in Fig. 2C, we found that 1) the LRP-1-depleted cells have completely lost self-supported motility under serum-free conditions (panel e vs. panel d) and 2) LRP-1 downregulation did not affect physiological stimulus-, i. e. serum-stimulated motility of the same cells (panel f). We confirmed this important finding using an independent cell migration assay, the “scratch” assay, which measures the relative and directed migration of a cell population. As shown in Fig. 2D, under serum-free conditions, migration of the 231-LRP-1-KD cells was drastically delayed (panels i and j), in comparison to 231-wt cells (panels g, h). It must be point out that a basal level of cell migration in “scratch” assay, instead of zero migration, was due to an intrinsic issue of this assay: natural diffusion by the crowded cell monolayer toward an open cell-free area. Computer-assisted quantitation of both motility assays is shown in Fig. 2E,F. Results of the quantitation demonstrated that the LRP-1 plays a critical role in self-supported motility of the tumour cells.

### The “eHsp90α > LRP-1 autocrine loop” only accounts for a portion of the self-supported tumour cell motility

**Figure 3:**
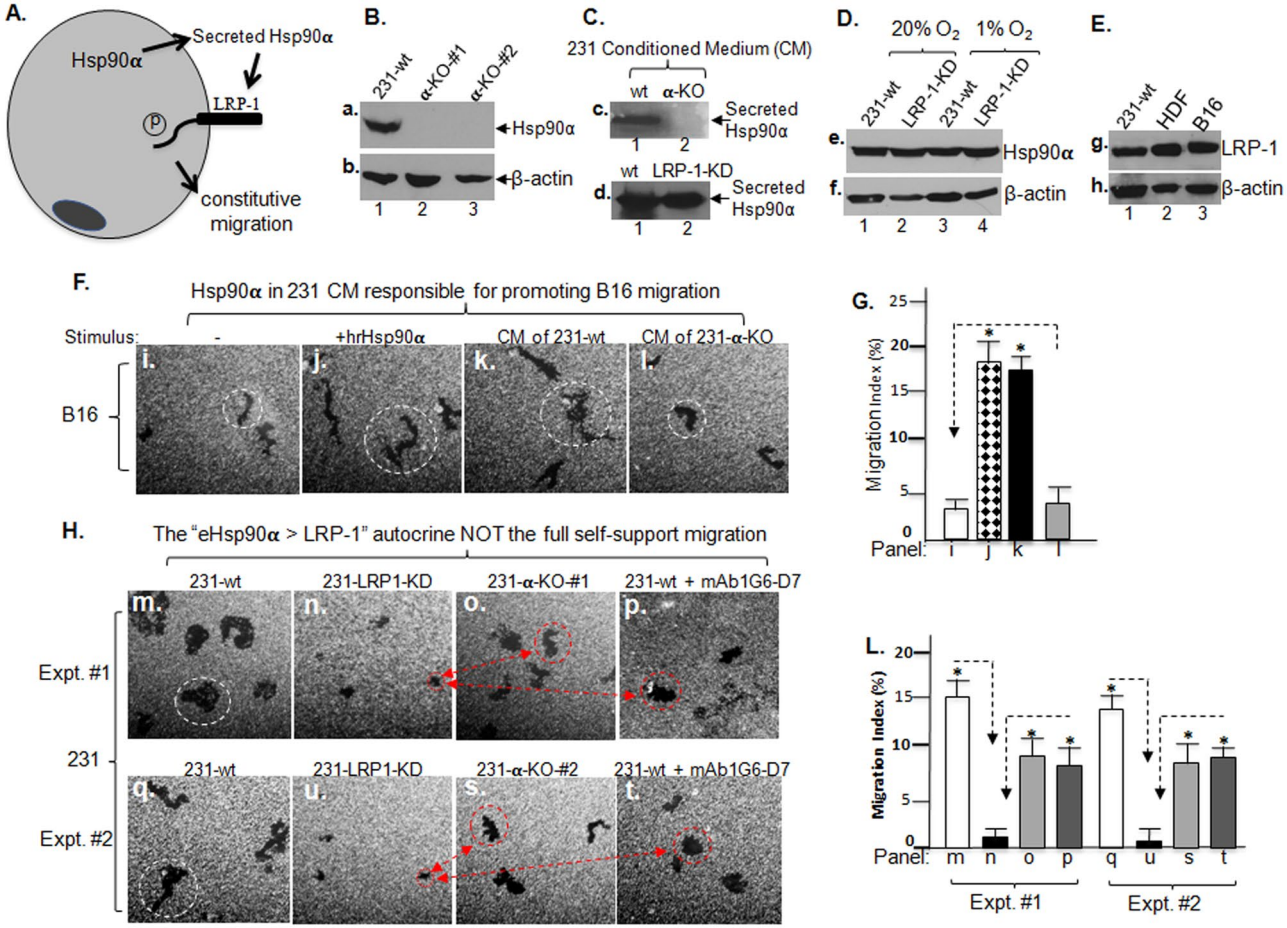
The “eHsp90α > LRP-1 autocrine loop” only accounts for 50% of the constitutive motility of the tumour cells. (**A**) A schematic illustration of the “eHsp90α > LRP-1 autocrine loop” that promotes cell motility. (**B**) Western blot shows 231-wt and two clones of 231-Hsp90α-knockout (231-α-KO) cells (panel a). (**C**) Anti-Hsp90α antibody blotting of secreted Hsp90α in serum-free conditioned medium (CM, 10X) from 231-wt, 231-α-KO (panel c) or 231-LRP-1-KD (panel d) cells. (**D**) Western blot analysis shows the total Hsp90α level in 231-wt or 231-LRP-1-KD cells under normoxia or hypoxia. (**E**) Western blot analysis shows the relative levels of LRP-1 in indicated cell lines. (**F**) CM from 231-wt, but not 231-α-KO, cells stimulated B16 melanoma cell migration (panels k and l vs. panel i), with human recombinant (hr) Hsp90α protein (panel j) as positive control. (**G**) Computer-assisted quantitation of the B16 cell migration shown in panel F as Migration Index (%) (“Methods”). CM from 231-α-KO cells completely lost pro-motility activity (panel l). Asterisks indicate significance of the induced migration by indicated stimuli (panels j, k) versus the serum-free control (panel i). (**H**) The indicated cells were simultaneously compared for their motility under serum-fee conditions. Neither total α-KO (panels o and s) nor extracellular blockade of secreted Hsp90α function by mAb1G7-D7 (panels p, t) re-produced the degree of inhibition of cell motility by LRP-1-KD (panels n and u), in reference to 231-wt cells (panels m and q). (**L**) Computer-assisted quantitation of the cell migration data with indicated panels. Asterisks indicate the significance of indicated cell motility over that of the LRP-1-KD cells. All experiments were repeated multiple times to reach the final conclusion.

To investigate the possible mechanism by which the LRP-1 receptor supports the constitutive motility of the tumour cells, we initially speculated that the previously identified “tumour-secreted Hsp90α > LRP-1 receptor” autocrine pathway^19,20^ as schematically shown in Fig. 3A, must be the candidate. To study this hypothesis, we focused on the serum-free conditioned medium (CM) of the 231-wt and 231-Hsp90α-KO (231α-KO) cells. Total lysates of two independent 231α-KO clones, α-KO-#1 and α-KO-#2, were blotted with anti-Hsp90α antibody (Fig. 3B, panel a), which we have previously generated using CRISPR-cas9 gene-editing technique^7^. Then, serum-free conditioned medium was collected from wild type and α-KO 231 cell. Since 231 cells already have a high basal motility due to the “secreted Hsp90α > LRP-1 receptor” autocrine^7,8^, we chose an independent target cell line, the B16 mouse melanoma cells that show a much lower basal cell motility, to test the CM of 231-wt and α-KO 231 cells. As shown in Fig. 3C, as expected, the Hsp90α protein was only detected in CM from 231-wt (lane 1), but completely absent from α-KO cells (lane 2). In contrast, secretion of Hsp90α remained unchanged in LRP-1-KD 231 cells (panel d, lane 2 vs. lane 1). Furthermore, the total cellular Hsp90α protein level remained unchanged between 231-wt and LRP-1-KD cells under either normoxia or hypoxia condition (Fig. 3D). Like 231 and human dermal fibroblast (HDF) cells, B16 cells showed LRP-1 expression positive (Fig. 3E) for justification as target cells for the CM test.

As shown in Fig. 3F, the CM from 231-wt cells strongly stimulated B16 cell migration (panel k vs. panels i). In contrast, the CM from the α-KO 231 cells completely lost the pro-motility activity (panel l vs. panel i). Human recombinant (hr) Hsp90α protein-stimulated migration was included as the positive control (panel j). These results suggest that secreted Hsp90α in the CM of 231-wt cells was a main stimulus of target cell migration. Computer-assisted quantitation of the migration data is shown Fig. 3G, clearly showing the critical role for secreted Hsp90α. However, to our total surprise, neither depletion of Hsp90α nor mAb 1G6-D7 neutralization of the secreted Hsp90α function from the extracellular environment could replicate the full effect of LRP-1 downregulation. As shown in Fig. 3H, in comparison to LRP-1 down-regulation (panel n), neither Hsp90α-KO, α-KO-#1 cell, (panel o) nor anti-Hsp90α neutralizing antibody, mAb1G6-D7, (panel p) was able to achieve a complete inhibition of the self-supported motility of the tumour cells. Similar results were obtained from an independent Hsp90α-KO cell clone, α-KO-#2, (panels q to t). The finding of two independent cell clones and mAb1G6-D7 was confirmed by data quantitation (Fig. 3L).

**Figure 4:**
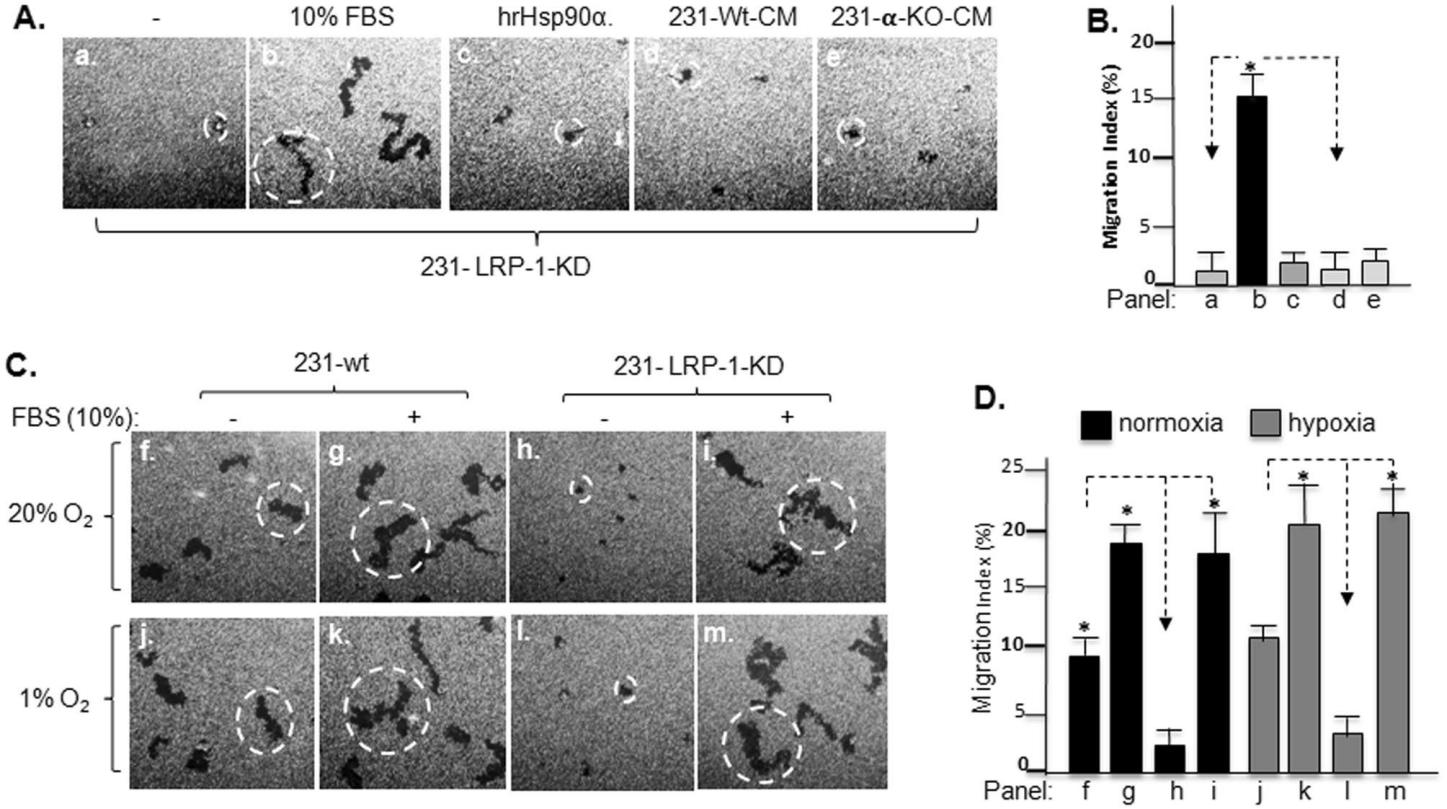
Only serum, but not 231-wt CM, hrHsp90α or hypoxia, rescues the blocked constitutive migration by LRP-1-KD. To further confirm that secreted Hsp90α in the “eHsp90α > LRP-1 autocrine loop” only presents a part of the tumour cells’ constitutive motility, all relevant extracellular factors were tested. (**A**) hrHsp90α protein (panel c), 231-wt CM (panel d) and 231-α-KO CM (panel e) all showed little effect, while serum (panel b) rescued as expected. (**B**) Computer-assisted quantitation of the migration data. (**C**) The indicated cell lines were serum-starved and subjected to colloidal gold migration assay un normoxia (20% O_2_) or hypoxia (1% O_2_) (a main driver of Hsp90α secretion^7^) without or with FBS (10%). Dotted circles indicate that the average size of the migration tracks under each condition. (**D**) Computer-assisted quantitation of the migration data shows limited rescue of LRP-1-KD cell motility by hypoxia (panel l vs. panel h).

To further substantiate the finding of the critical role for secreted Hsp90α instead of other factors in the CM, as shown in Fig. 4A, we found that CM from either 231-wt (panel d) or 231-α-KO (panel e), as well as hrHsp90α protein (panel c), were all unable to promote migration of the 231-LRP-1-KD cell, whereas the cells still achieved full migration under serum stimulation (panel b). Computer-assisted quantitation of these migration data is shown in Fig. 4B. Finally, on the 231-LRP-1-KD cells, we found that hypoxia showed little rescuing effect. As shown in Fig. 4C, LRP-1 downregulation blocked 231 cell migration (panel h vs. f). Hypoxia had little effect on the results (panels j and l), whereas the cells responded fully to serum as expected (panels g vs. i and panels k vs. m). Quantitation of these data shown is presented in Fig. 4D, which indicates that hypoxia’s specific contribution to tumour cell constitutive motility is limited. Taken together, the above data indicate that i) secreted Hsp90α is the main pro-motility factor from CM of 231 tumour cells and ii) the secreted Hsp90α > LRP-1 autocrine only partially accounts for the constitutive motility of the tumour cells.

### LRP-1 stabilizes activated EGFR that accounts for the second pro-motility pathway

**Figure 5:**
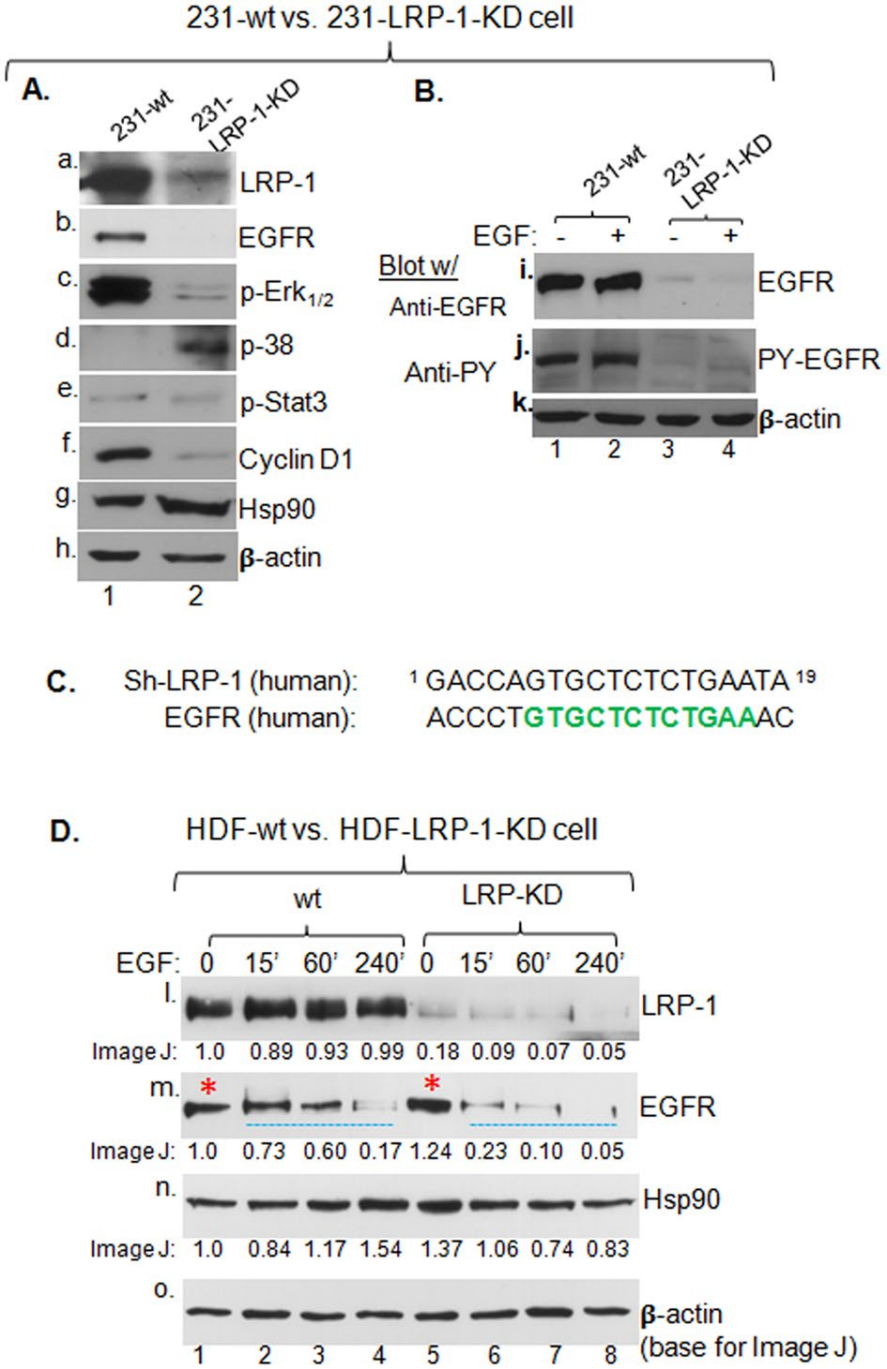
LRP-1 downregulation destabilizes activated EGFR in both tumour and normal cells. (**A**) Total lysates of 231-wt and 231-LRP-1-KD cells were immunoblotted with indicated antibodies to show that downregulation of LRP-1(panel a) caused disappearance of EGFR (panel b) and affected known EGFR downstream targets, but not other controls (panels c to g). (**B**) Total cell lysate of the two cell lines with or without hrEGF (100 ng/ml) stimulation for 2 min were blotted with either anti-EGFR protein (panel i) or anti-phosphotyrosine (PY) (panel j) antibody. (**C**) The best continuous nucleotide sequence match between the target sequence of shRNA against LRP-1 and the human EGFR cDNA is shown. (**D**) Primary human dermal fibroblasts with or without down-regulated LRP-1were stimulated with hrEGF (100 ng/ml) for indicated times. O.D. equalized total cell lysates were subjected to Western immunoblotting analysis with indicated antibodies. The intensity of the signals was measured by Image J, based on their corresponding β-actin band signals. Asterisks indicate inactivated EGFR proteins, indicating no cross reactivity from the shRNA-LRP-1.

The previous finding suggested that, besides the “secreted Hsp90α > LRP-1 receptor” autocrine pathway, LRP-1 regulates another independent signalling pathway to achieve a full control of the self-supported tumour cell motility. To identify the second pathway, we carried out antibody screening for stability of the major signalling molecules in the LRP-1-downregulated 231 cells and made a surprising finding. As shown Fig. 5A, the downregulation of LRP-1 (panel a, lane 2) correlated with disappearance of the EGFR (epidermal growth factor receptor) (pane b, lane 2), as well as reduced levels of two EGFR downstream effectors, the phosphorylated Erk1/2 (panel c, lane 2) and the G1 phase cyclin D1 (panel f, lane 2). Whereas other non-direct downstream targets either remained unchanged, such as p-Stat3 (panel e), or increased, such as p38 (panel d) and total Hsp90 (panel g) protein. Consistently, under our experimental conditions, the majority of EGFR in 231 cells is constitutively activated even under serum-starved conditions and EGF stimulation only slightly further enhanced its activation, as indicated by anti-phosphotyrosine (PY) antibody blotting (Fig. 5B, panel j, lanes 1 vs. 2) and LRP-1-downregulation correlated with disappearance of constitutively activated EGFR (panels i and j, lanes 3 and 4), the direct upstream activator of Erk1/2 and cyclin D1. These data suggest that LRP-1 regulates, via some unknown mechanism, the stability of activated EGFR.

One could question if the shRNA against LRP-1 cross reacted with the EGFR in the same cells. To this argument, we present here the 19-nucleotide shRNA sequence, designed by the Synthego CRISPR Design Tool (https://www.synthego.com/products/bioinformatics/crispr-design-tool) that minimizes cross reactivity of an shRNA, in alignment with the human EGFR cDNA that encodes shRNA-targeting EGFR mRNA. First, as shown in Fig. 5C, there are 12 continuous nucleotide sequence (in green) from human EGFR gene that matched 12 of the 19-nucleatide shRNA against human LRP-1 gene. Second, nonetheless we wanted to provide direct evidence for our findings that i) our shRNA against LRP-1 does not cross react with EGFR and ii) LRP-1 downregulation selective weakens the activated EGFR stability. To achieve the two goals at once, we took the advantage of primary human dermal fibroblast (HDF) cells that express inactive EGFR under serum-free conditions and exhibit EGF-stimulated tyrosine-phosphorylation of EGFR in a time-dependent fashion. Thus, we compared the stability of the EGFR between parental and LRP-1 downregulated HDFs without or with EGFR stimulation over time. As shown in Fig. 5D, the shRNA-mediated downregulation of LRP-1 was near complete (panel l, lanes 5–8 vs. lanes 1–4), with quantitation of the band intensities in reference to the β-actin’s by Image J shown underneath. Under these conditions, it is clearly shown that there was little cross reactivity of the shRNA against EGFR (panel m, lane 5 vs. lane 1, with an asterisk mark in red), instead a slight increase in EGFR. However, the EGF-activated EGFR declined significantly faster in LRP-1-KD cells (lanes 6–8) than the parental cells (lanes 2–4) with at least a three-fold difference based on the Image J data. The Hsp90 (panel n) and β-actin (panel o, the basis for Image J) were included as controls.

### Simultaneous inhibitions of both eHsp90 > LRP-1 and EGFR signalling pathways completely eliminate the self-supported motility of the cancer cell

**Figure 6:**
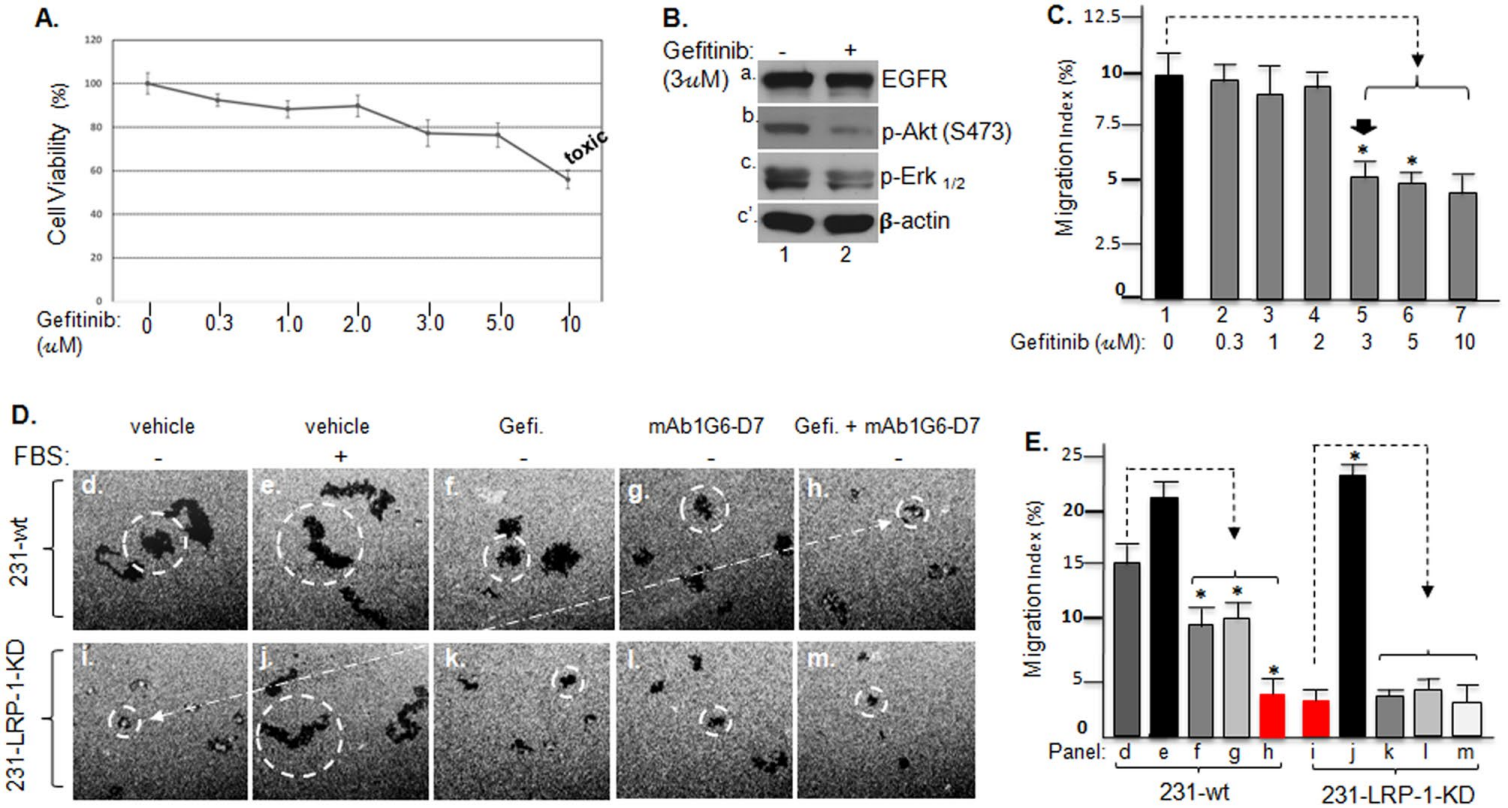
LRP-1-supported EGFR signalling adds the second 50% of constitutive motility of the tumour cells. (**A**) The toxicity profile of the EGFR inhibitor, Gefitinib, in 231 cell survival (“Methods). (**B**) Western immunoblotting analysis shows the effectiveness of Gefitinib to block Akt and Erk phosphorylation, direct downstream effectors of EGFR activation (panels b and c). (**C**) Cell migration assay shows the maximum inhibition by Gefitinib at the non-toxic concentration of 3 μM, as quantitated by computer-assisted calculation as Migration Index (%). Asterisks indicate significant inhibition of the cell migration by Gefinitib versus the control (bar 1). (**D**) Images of colloidal gold migration assay of 231-wt and 231-LRP-1-KD cells under indicated conditions. Dotted line points out that simultaneous inhibitions of “secreted Hsp90 > LRP-1 autocrine loop” by mAb1G6-D7 and EGFR signalling by Gefinitib (panel h) reproduces the effect of LRP-1 downregulation (panel i) on constitutive motility of the tumour cells. (**E**) Computer-assisted quantitation of 15 randomly selected microscopic images from each experimental condition in D as Migration Index (%). These results were confirmed by three independent experiments. Asterisks indicate significance of inhibition of cell migration by Gefinitib (bar f), mAb1G6-D7 (bar g) or both (bar h) versus their corresponding control (bar d). In contrast, no significant difference between bar h and bar I (in red).

We speculated that LRP-1 is at the pivotal position to coordinate both the “eHsp90α > LRP-1” autocrine signalling and the EGFR signalling to support the constitutive motility of the cancer cells under serum-free conditions. First, we tested the role of the activated EGFR in the self-supported motility of 231 cells by using the EGFR tyrosine kinase-specific inhibitor, Gefitinib (C_22_H_24_ClFN_4_O_3_). As shown in Fig. 6A, while most previous studies recommended 5 μM or higher concentrations of Gefitinib, we chose 3 μM as our working concentration, which dramatically reduced the phosphorylation of Akt and Erk_1/2_, two key downstream targets of activated EGFR, without significant cell death (Fig. 6B, panels b, c lane 2). Please note that the cell number without Gefitinib (“0”) included a small portion of proliferated cell after 16 h of incubation, i. e. slightly higher than the starting/seeding cell number on day 0. As expected, Gefitinib was only able to block approximately half of the self-supported motility of the tumour cells, prior to significant cell death by 10 μM Gefitinib (Fig. 6C). Therefore, to test whether simultaneous inhibitions of both the “eHsp90α > LRP-1 receptor autocrine loop” and the EGFR signalling would reproduce the effect of LRP-1 downregulation, we carried out experiments to compare single and double inhibitions in both 231-wt and 231-LRP-1-KD cells. As shown Fig. 6D, 231-wt cells exhibited constitutive motility even in the absence of serum (panel d) and serum further enhanced the motility (panel e). In contrast, 231-LRP-1-KD cells completely lost constitutive motility in the absence of serum (panel i), but serum was still able to promote the cell motility similar to what it did to 231-wt cells (panel j vs. panel e). Gefitinib (panel f) or mAb1G6-D7 (panel g) only partially blocked the self-supported motility of the tumour cells. However, the addition of both Gefitinib and mAb1G6-D7 showed additive effect on inhibition of the tumour cell migration (panel h), similar to the LRP-1 downregulated cells (panel i). As expected, neither the EGFR inhibitor nor the anti-eHsp90α antibody alone or in combination could further affect the motility of the 231-LRP-1-KD cells (panels k, l, m vs. panel i). Computer-assisted quantitation of the migration data is shown in Fig. 6E, which clearly indicate that the tumour cells utilize LRP-1 to coordinate both eHsp90α signalling and EGFR signalling for maintaining self-supported motility under serum-starved conditions.

## Discussion

**Figure 7:**
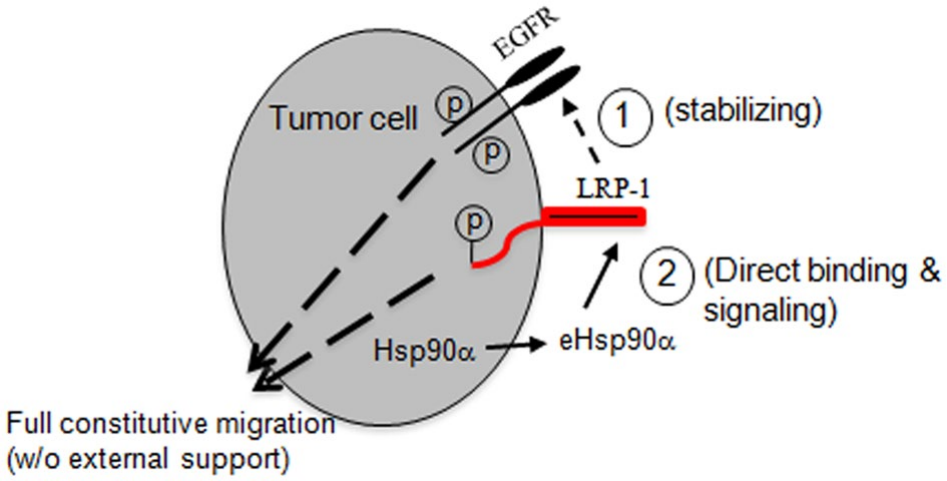
A working model for LRP-1-coordinated mechanism for constitutive motility of MDA-MB-231 tumour cells. Migration and invasion of different tumours are likely driven under distinct tumour driver genes. Therefore, the current finding represents a mechanism that regulates the constitutive motility of certain tumour types.

Tumour cell self-supported motility serves as a foundation for tumour invasion and metastasis, which kill patients. Similar to the findings that different oncogenes and tumour suppressor mutant genes drive tumorigenesis of different types of tumours, the mechanisms behind tumour cell self-supported motility likely vary among different tumours and currently remain largely understudied. Understanding the mechanisms could guide the designs of target-specific therapeutics or even personalized anti-tumour drugs. In the current study, we have taken the highly malignant and well-characterized human triple negative breast cancer cell lines, MDA-MB-231 (231), as the cell model to report the first mechanism that drives self-supported tumour cell motility under serum-free conditions, a characteristic microenvironment during tumorigenesis. In contrast to the conventional wisdom of mutated oncogene-driven mechanism, our study has revealed a parallel dual signalling pathway-participated mechanism with the cell surface LRP-1 as the central coordinator. As schematically depicted in Fig. 7, LRP-1 receptor maintains stability of tyrosine-phosphorylated EGFR signalling at one hand and mediates the tumour cell-secreted eHsp90α autocrine signalling on the other hand, to achieve a full self-supported motility that reaches about 60% of the full cell motility under physiological conditions. We suggest that in the absence of sufficient serum support, the “starved” tumour cells constantly depend upon this self-supported migration to move away from the (temporal) hazard environment of no blood support until they gain new environmental support.

The finding that LRP-1 maintains the stability of activated EGFR cells is not cell type-specific. Similarly, another highly related breast cancer cell line, MDA-MB-468, has an undetectable level of LRP-1 and yet a much higher level of inactive EGFR^7^. Moreover, EGFR in primary human dermal fibroblasts under serum starvation remains inactivate, unless EGF is added to the medium. We showed that down-regulation of LRP-1 had little effect on the inactivated EGFR in the absence of EGF stimulation. However, the level of EGF-stimulated EGFR declined dramatically faster in LRP-1-downregulted cells (see Fig. 5D). Currently, how LRP-1 maintains the stability of active EGFR remains unknown. Since the full-length cDNA for LRP-1, which encodes for a 515-kDa extracellular α subunit and an 85-kDa trans-membrane β subunit, exceeds the maximum cloning limit for any cDNA expression vectors^21^, it is technically not possible to do rescue experiments with the full-length LRP-1 cDNA. Our preliminary study show that none of the four mini-LRP-1 receptor cDNAs that encodes the transmembrane 85-kDa β subunit plus one of the four extracellular domains (I to IV)^22^ was able to stop the faster downregulation of EGF-stimulated EGFR. These results suggest that combined extracellular domains or even the full-length LRP-1 is required for stabilizing activated EGFR at cell surface. A more exciting possibility is what role for LRP-1 to play in stability of tyrosine kinase receptors in general. So far, Takayama et al. reported that LRP1 controls endocytosis and c-CBL-mediated ubiquitination of the platelet-derived growth factor receptor β (PDGFRβ)^23^. Gopal and colleagues showed that the eHsp90-LRP1 signalling axis involves activation of the EphA2 tyrosine kinase receptor^14^. Together with our current finding of LRP-1 on active EGFR, it is of a great interest to further explore the broader possibility that LRP-1 serves as a “mother co-receptor” for all tyrosine kinase receptors.

Independent of the EGFR signalling, the second parallel “eHsp90α > LRP-1 autocrine loop” to promote tumour cell migration has been clearly established by previous studies^8,13,19,22^. Normal cells secrete eHsp90α only under stress, such as tissue injury signals, whereas many tumour cells have acquired the ability to constitutively secrete Hsp90α driven by oncogenes, such as overexpressed HIF-1α and mutant p53^8,24,25^. The sole function of eHsp90α is to promote cell motility during wound healing, which is taken advantage of by tumour cell invasion. Secreted Hsp90α acts as a *bona fide* signalling protein that binds to the subdomain II in the extracellular part of LRP-1 and activates, via the NPVY motif in the cytoplasmic tail of LRP-1, the Akt and Erk1/2 kinases^22^. In all, this current study is the first report on a novel mechanism that supports tumour cells’ constitutive motility, a prerequisite for tumour invasion and metastasis.

## Materials and methods

### Cell lines, antibodies and reagents

Limited-passage stocks of the human triple negative breast cancer cell line, MDA-MB-231 (231), and non-transformed breast epithelial cells, HBL-100, were periodically obtained from liquid nitrogen storage. Hsp90α-knockout 231 cells (231-α-KO) were established by CRIPR-cas9 gene-editing technique, as described previously^7^. Primary human dermal fibroblasts (HDFs) were purchased from Clonetics and cultured in DMEM supplemented with 10% FBS and 1% Penicillin–Streptomycin (Gibco/Invitrogen Corp, Carisbad, CA). B16 moue melanoma cell line and all other cells were cultured in DMEM medium with high glucose supplemented with 10% FBS (Thermo Scientific, MA, USA), prior to serum-starvation (0.3% serum) and experiments. 293 T cells for lentivirus production were cultured in DMEM with high glucose and 10% FBS (Thermo Scientific, MA, USA). Mouse monoclonal antibody against the p85 subunit of LRP-1/CD91 was purchased from Invitrogen Co (#37-7600, Grand Island, NY). Anti-EGFR antibody (D38B1, #4267) and anti-LRP-1 antibody (#64099) were from Cell Signalling Technology (Beverly, MA). EGFR tyrosine kinase inhibitor, Getifinib (SML 1657) was purchased from Sigma-Aldrich. Anti-phosphotyrosine antibody (#72) was a gift from Dr. Joseph Slessinger (Yale University). Anti-Hsp90α antibody purchased from Calbiochem (NB120-2928, Novus Biologicals, CO). The mouse monoclonal antibody, mAb1G6-D7, which neutralizes secreted Hsp90α function, was established in our laboratory^10^. Anti-phospho-Smad3, anti-phospho-p38 kinase, anti-phospho-p44/42 MAPK (D13.14.4E, #4370) from the Cell Signalling Technology (Beverly, MA), anti-Cyclin D1 antibody (EPR2241, GTX61845, GeneTex, Irvine, CA) and anti-β-actin antibody (AC038, Transduction Laboratories, San Jose, CA) were as indicated. ECL western blotting detection reagent (# RPN2106, Amersham, Inc., Marlborough, MA).

### Lentivirus production, infection and Western blot analysis

The selected shRNA sequence (sense) against human LRP-1/CD91, GACCAGTGCTCTCTGAATA, was cloned into lentiviral vector, FG12, for delivering shRNA was as previously described^8,19^. This construct was mixed with two packaging vectors, pCMVΔR8.2 and pMDG, and used to transfect 293 T cells to produce virus stocks for infection of target cells, such as 231 and HDF cells. Following infection, total cell lysates of different treatments were equalized using BCA Protein Assay Kit (Thermo Scientific). Protein samples were resolved in SDS-PAGE, transferred onto a nitrocellulose membrane and stained with Ponceau S solution to confirm efficient and even protein transfer. The membrane was blocked by 5% BSA (in TBS buffer) prior to incubation with primary antibodies against as indicated. Secondary anti-rabbit IgG (1:10,000) and anti-mouse IgG (1:10,000) were used as instructed by manufacturers, followed by ECL reaction. Protein bands from the same blot of the same experiment was quantitated using the NIH Image J software^7,22^.

### Crystal violet staining of live cells

50% confluence cells were serum starved for one day or two days, respectively according to the cell migration protocols. Cells were rinsed with PBS three times to remove dead cells and the attached cells were stained with 0.4% crystal violet solution in 20% methanol for 10 min at room temperature. The plates were washed three times with water and inverted on filter paper for air dry at room temperature. Adherent cells were imaged at 10 × magnification using a light microscope. Randomly selected 5 images (2 mm × 1.4 mm each) for each condition under the microscope were recorded. Cell number within 0.3mm^2^ was counted for each recorded image. The cell numbers from the 5 images per condition were averaged to reflect the cell survival rate.

### Cell survival and growth assay

Cells during their exponential growth phase were used for this assay. Cells were re-seeded at 8 × 10^4^ cells/well in 12-well plate. The attached cells in triplicates were lifted by trypsin and cell number counted as day 0 start point (without treatments). The medium was replaced with complete medium without or with indicated concentrations of Gefitinib and incubated for 48 h. The number of viable cells in each well was counted and averaged numbers from triplicates per concentration plotted as number of cells versus increasing concentrations of Gefitinib. All numerical results are reported as mean and standard deviation (s.d.).

### Colloidal gold cell motility assay and “scratch” cell migration assay

Cell motility colloidal gold phagokinetic assay with computer-assisted quantitation was as previously invented by Albrecht-Buehler and modified by our laboratory^16,17^. Briefly, bovine serum albumin (BSA, 1%) -coated coverslips (35 mm in diameter) were placed into 12-well tissue culture plates with one coverslip per well. Gold salt solution (9% of gold salt is combined with 52% of H2O and 30% of the Na2CO3 solution) was heated in a 50 ml Erlenmeyer flask with constant swirling until boiling, removed from the heat source. An equal volume of freshly prepared formaldehyde (0.1%) was slowly added to the gold salt mixture with gentle swirling. After the mixture turned brown, the solution was immediately pipetted into the 12-well plates with BSA-coated coverslips at 1 ml per well. The plates were covered and left undisturbed for at least 2 h to settle gold salt particles. Following removal of the upper liquid solution, the coverslips were gently rinsed once with 1 ml of Hank's buffered salt solution (HBSS) and coated with type I collagen in HBSS at 37 °C for 2 h. After free collagen was removed and plates were rinsed once with HBSS, 3,000 cells were plated into onto each well with indicated conditions and allowed to migrate for 16 h. Cell migration was examined under dark field microscope linked to a computer to visualize on screen. Fifteen randomly selected and nonoverlapping fields under each experimental condition were analyzed with an attached CCD camera (Model KP-MIU, Hitachi-Denshi) and a computer using the NIH Image 1.6 program, which calculates migration index (MI), which represents the percentage (%) of the cell migration tracks-consumed area over the total field area viewed by the microscope.

A modified procedure of the in vitro wound-healing (“scratch”) assay was performed according to Li and colleagues^17^. The main modifications include: (1) coating the surface of the tissue culture wells with a specified ECM, e. g. collagen, fibronectin and (2) the wounds/scratches are made shortly after the cells fully attached (within 2–3 h following seeding) and floating cells removed. The pre-coated ECM and the relatively short cell attachment period prevented additional ECM deposition on the migratory surface contributed by the cells themselves. Mitomycin C (10 μg/ml) was included throughout the experiments to prevent cell proliferation. Each condition has triplicate wells for quantitation. Quantitation was made based on the (left) open cell-free area, in reference to 100% on day 0.

### Statistical analysis

Statistical significance was determined using a two-tailed Student’s t-test and one-way ANOVA. Final presentation as mean ± s.d. was based on at least three independent and corroborating experiments. Confirmation of a difference in migration as statistically significant requires rejection of the null hypothesis of no difference between mean migration indices obtained from replicate sets. The *p* value equal or less than 0.05 was considered statistically significant.

## Supplementary Information

Supplementary Information 1.

Supplementary Information 2.

Supplementary Information 3.

Supplementary Information 4.
