# Supplementary figures and images for: LRP-1 receptor combines EGFR signalling and eHsp90α autocrine to support constitutive breast cancer cell motility in absence of blood supply
Source: Sci Rep. 2022 Jul 14;12:12006. doi: 10.1038/s41598-022-16161-y (PMC9283467; doi:10.1038/s41598-022-16161-y)

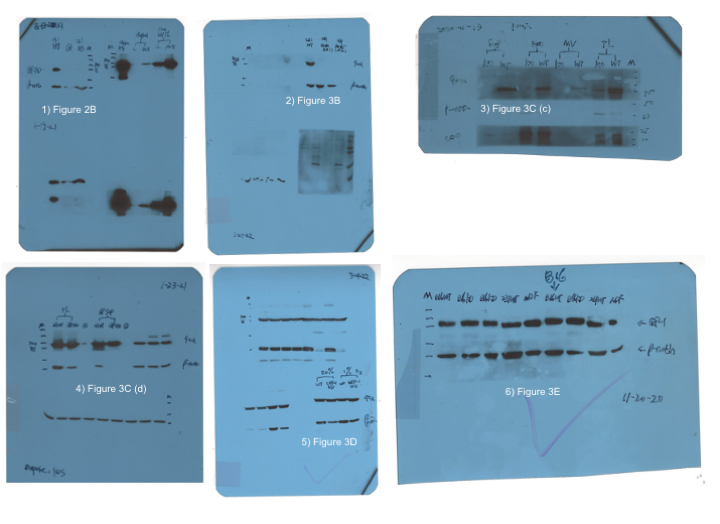

Supplement: Supplementary file 1 — Supplementary Information 1. [file 41598_2022_16161_MOESM1_ESM.tiff]

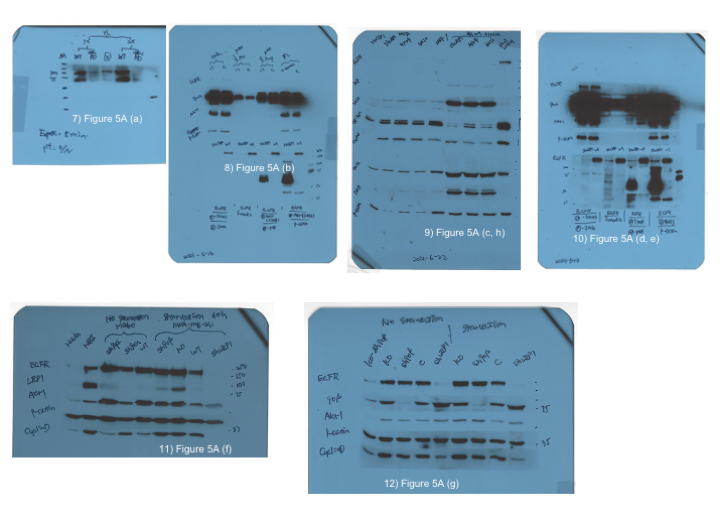

Supplement: Supplementary file 2 — Supplementary Information 2. [file 41598_2022_16161_MOESM2_ESM.tiff]

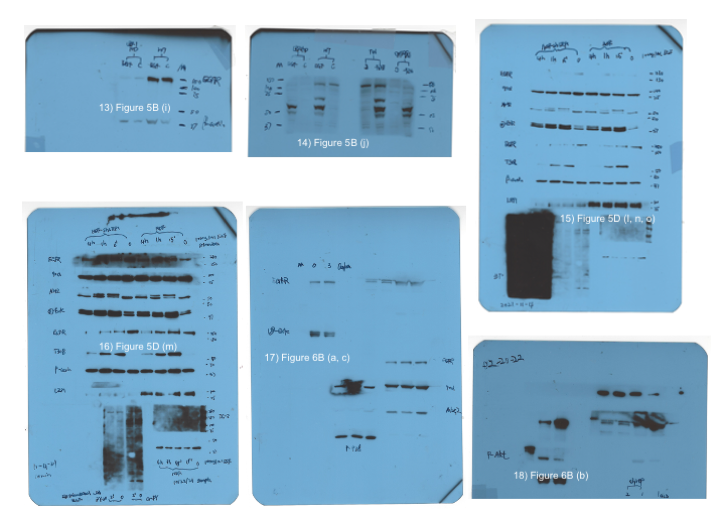

Supplement: Supplementary file 3 — Supplementary Information 3. [file 41598_2022_16161_MOESM3_ESM.tiff]

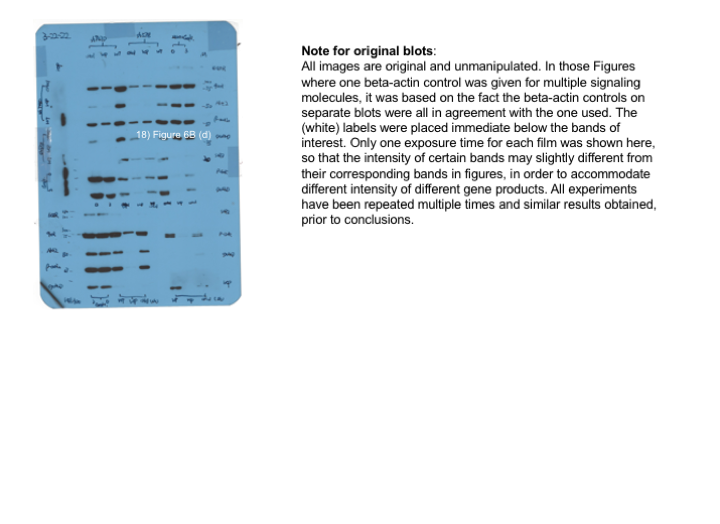

Supplement: Supplementary file 4 — Supplementary Information 4. [file 41598_2022_16161_MOESM4_ESM.tiff]
